# Unusual presentation of advanced prostate cancer in a black population of South-Western Nigeria

**DOI:** 10.11604/pamj.2019.32.15.14417

**Published:** 2019-01-10

**Authors:** Abdulkadir Salako, Tajudeen Badmus, Akinwunmi Komolafe, Rotimi David, Martin Igbokwe, Adeyinka Laoye, Ibrahim Akinbola, Rereloluwa Babalola, Chigozie Onyeze

**Affiliations:** 1 Urology Unit, Department of Surgery, Obafemi Awolowo University Teaching Hospitals Complex, Ile-Ife, Nigeria; 2 Department of Morbid Anatomy and Forensic Medicine, Obafemi Awolowo University Teaching Hospitals Complex, Ile-Ife, Nigeria

**Keywords:** Prostate cancer, unusual presentation, South-western Nigeria

## Abstract

There are growing concerns on the varying pattern of advanced prostate cancer (PCa) presentation across the world. We report some of the unusual presentations of PCa at the Obafemi Awolowo University Teaching Hospitals Complex (OAUTHC), Ile-Ife, South-Western Nigeria. A review of all patients with histologically confirmed PCa who had unusual presentations between January 2014 and December 2015 was done. Unusual presentation was defined as an atypical feature in the absence of lower urinary tract symptoms (LUTS), with the diagnosis of PCa only suspected after abnormal digital rectal examination (DRE) and/or elevated prostate specific antigen (PSA) assay. Thirteen patients had an unusual presentation in OAUTHC during the study period. Five (38.5%) had left supraclavicular swellings while four (30.8%) had haematochyzia and tenesmus. Other unusual presentations include large bowel obstruction requiring emergency colostomy (2;15.4%) and a scalp mass (1;7.7%). All patients had appropriate treatment for stage of PCa and are being followed up in the out-patient clinic. The change in presentations of PCa may suggest the need for DRE and serum PSA assay among all middle-aged and elderly men presenting at health facilities. Large scale studies on PCa across different population groups may also help at identifying related clinical, demographic and epidemiological factors as well as possible validation of some of these unusual presentations.

## Introduction

Prostate cancer (PCa) is a global health burden [1, 2]. It is the most common male malignancy worldwide and the second major cause of male cancer deaths. The incidence however varies widely among populations [1]. The highest incidence and mortality has been recorded in migrant and native black sub-populations worldwide and it is generally termed the malignant epidemic of the blacks [3, 4]. In the past, several authors reported a low incidence of prostate cancer in Africa, including Nigeria. Recently however, reports from Southern Nigeria have shown a high and rising incidence of the disease [1]. Its aetiology is largely conjectural. There is no PCa screening policy yet in Nigeria and majority of the patients present with late disease. Osseous and visceral metastatic presentation is known among these patients [1, 5, 6]. Documentation on the unusual extra-urinary tract sites is however scanty in our practice in South-Western Nigeria.

## Methods

Data of men referred to the Urology Unit between January 2014 and December 2015 who presented with extra-urinary tract features without lower urinary tract symptoms, suspected to have cancer of the prostate from abnormal digital rectal examination (DRE) findings and/or elevated prostate specific antigen (PSA) were reviewed. The information obtained was analysed for age, presenting features, DRE findings, PSA levels, biopsy results, treatment and treatment outcome.

## Results

**Figure 1:**
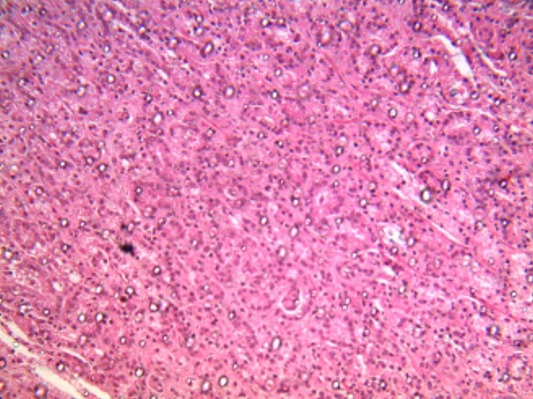
Microscopic appearance of prostatic carcinoma: the field shows micro-acini of small malignant cells infiltrating the prostatic stroma in areas (H & E x 100)

**Figure 2:**
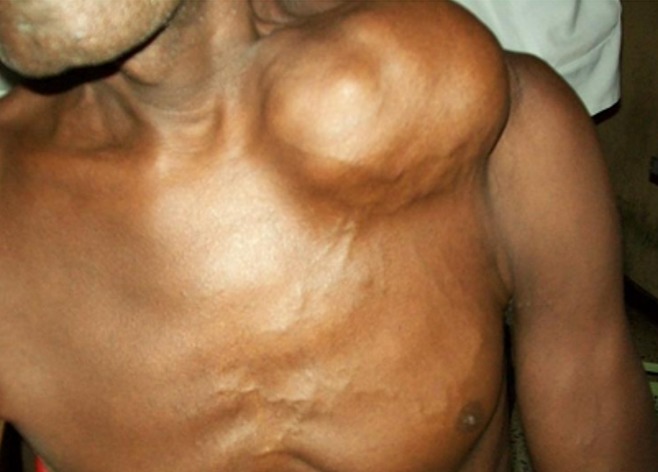
Huge left supraclavicular mass

**Figure 3:**
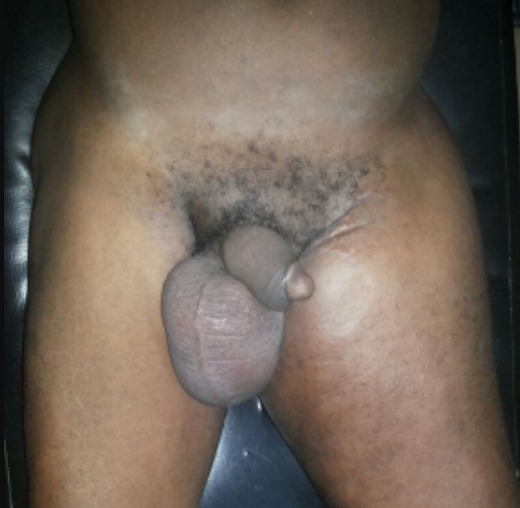
Penile, scrotal and bilateral lower limb lymphoedema + inguinal lymphadenopathy

**Figure 4:**
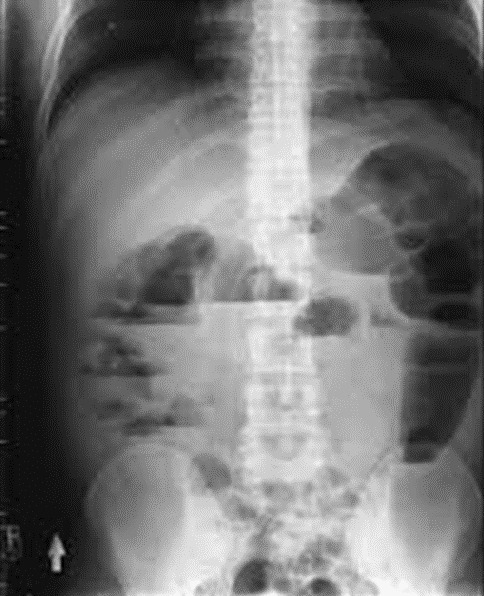
Erect plain abdominal Xray showing multiple air-fluid levels from advanced PCa

**Figure 5:**
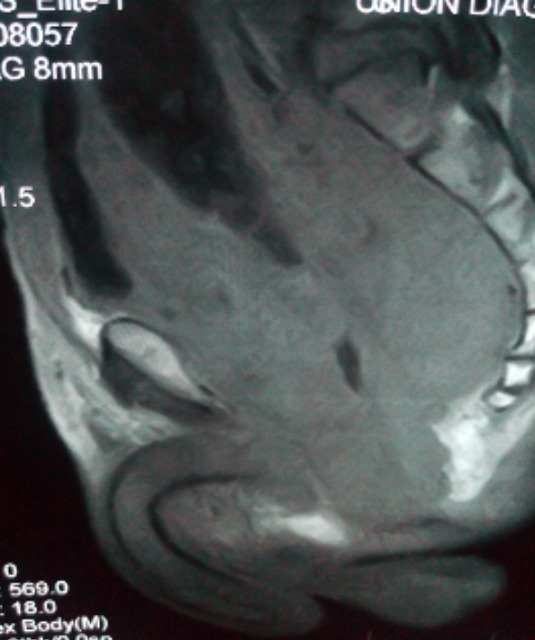
Lateral pelvic Xray showing large bowel obstruction from PCa

**Figure 6:**
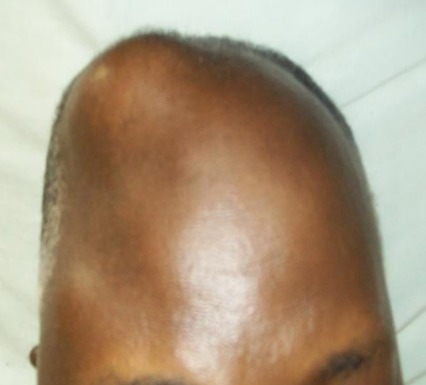
Right frontal mass

**Figure 7:**
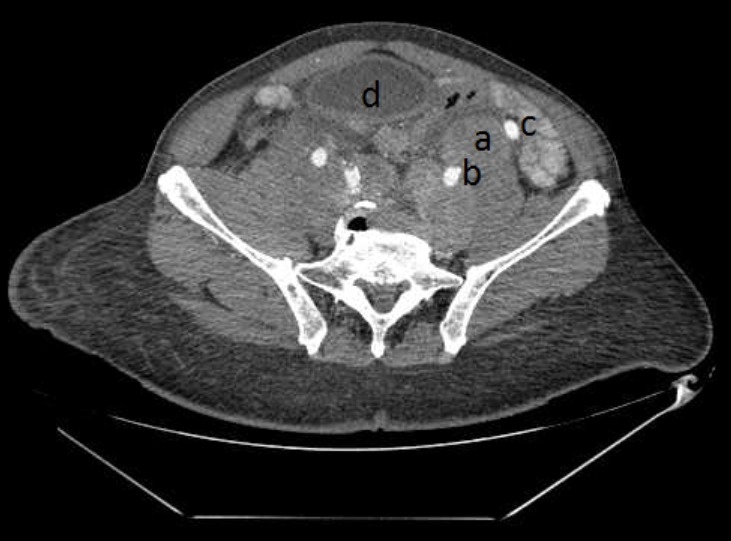
Contrast enhanced pelvic CT scan showing (a) a huge heterogenous dense structure (prostate gland) indenting the posterior bladder wall

Thirteen patients had unusual PCa presentation in OAUTHC during the study period. Prostatic adenocarcinoma was the histopathological finding in all (100%) these patients (Figure 1). Five (38.5%) had supraclavicular swelling (Figure 2) of which two (15.4%) of them had additional left lower limb, scrotal, penile swelling and bilateral inguinal adenopathy (Figure 3); while 3 patients (23.1%) had haematochyzia and tenesmus. Other unusual presentations include acute large bowel obstruction (Figure 4, Figure 5) necessitating emergency colostomies (2; 15.4%), unexplained anemia (1; 7.7%), a scalp mass (1; 7.7%) (Figure 6) and a large retroperitoneal mass(1; 7.7%) (Figure 7). All had bilateral total orchidectomy (BTO) as part of their palliative care with satisfactory outcome.

### Patients' characteristics

**Supraclavicular masses (5 patients):** age: 59, 62, 63, 67, 68 years, mean 63.8yrs; PSA: 62, 978, 22, 25, 7.9 ng/mL respectively. Mean 218ng/ml; DRE: abnormal in all; prostate biopsy: infiltrating adenocarcinoma (Gleason 5-8); Treatment: - excision of lesion + BTO (Figure 2).

**Hematochezia and tenesmus (3 patients):** age: 56, 58, 60 years; mean 58yrs; PSA: 20, 12, 4.42ng/mL respectively. Mean 18.21ng/ml DRE: abnormal; prostate biopsy: infiltrating adenocarcinoma (Gleason 8- 10); treatment: blood transfusion, BTO (Figure 5).

**Scalp mass (1 patient):** age: 69 years; PSA: 44ng/mL; DRE: abnormal; prostate biopsy: infiltrating adenocarcinoma (Gleason 7); treatment: BTO (Figure 6).

**Large bowel obstruction (2 patient):** age: 81 years; PSA: 45 ng/mL; DRE: abnormal; prostate biopsy: infiltrating adenocarcinoma (Gleason 7,9); treatment: colostomy + BTO (Figure 4).

**Unexplained anemia (1 patient):** age: 79 years; PSA: 7.9ng/mL; DRE: abnormal; prostate biopsy: infiltrating adenocarcinoma (Gleason 6); treatment: blood transfusion, BTO.

**Large retroperitoneal mass (1 patient):** age: 79 years; PSA: 130mg/ml; DRE: abnormal; prostate biopsy: infiltrating adenocarcimona (Gleason 9); treatment: BTO (Figure 7).

## Discussion

Direct spread of prostate adenocarcinoma involving the seminal vesicle and bladder neck producing lower urinary tract symptoms and osseous metastasis from haematogenous route are the most common forms of spread in previous reports from the region [1, 5, 6]. Non osseous involvement of the scalp, supraclavicular sites and bowel involvement, causing rectal bleeding and/or intestinal obstruction are uncommon [6, 7]. All the patients reviewed presented in their sixth to eight decade of life with locally advanced and metastatic disease which is the more common pattern in late presentation [1]. The mean PSA for the patients was 115.03ng/ml with an average gleason score of 6.5 which are indicators of advanced disease and poor prognosis. This is prevalent in our environment [5, 8]. There seems to be a rising incidence of these atypical presentations of PCa when findings in this study are compared with local and international literature [5]. Reasons attributed to this include increasing awareness by physicians and patients on prostate cancer; and increasing availability of PSA screening centres [6]. There is however no policy yet on screening for prostate cancer in Nigeria and this may in addition explain the observed late presentation by these patients. Two patients presented with clinical features of large bowel obstruction while another patient presented with gastro-intestinal bleeding. Exploratory laparotomy for one of the patients with intestinal obstruction revealed an infiltrating mass at the recto-sigmoid junction which on resection and pathological examination showed poorly differentiated adenocarcinmona. PSA staining of the tissue was not available at that time. Elevated serum PSA and abnormal DRE in this patient prompted prostate biopsy which revealed prostate cancer, both patients had an emergency diverting colostomy as initial treatment.

The patient with lower gastro-intestinal bleeding had colonoscopy and a bleeding rectal mass was detected and biopsied. These patients showed remarkable improvement with androgen deprivation therapy (ADT). Involvement of the bowel in prostate cancer is rare and presents a diagnostic challenge [7, 9]. The fascia of denonvelliers protects the colon and rectum from PCa, however, when this fascia is penetrated in unusual situations, the tumour invades the rectum. These patients were managed by blood transfusions, emergency colostomy and ADT. Chang *et al* reported an obstructing recto-sigmoid tumour from metastatic PCa which was similarly treated with a good outcome [7]. Five of the twelve patients presented with a painless left supraclavicular swelling to the Cardio-thoracic Surgeons. Initial presentation of advanced PCa with supraclavicular swelling is uncommon [10]. Biopsies of these neck masses revealed metastatic adenocarcinoma which necessitated referral to the urology unit. All the patients had elevated PSA and abnormal DRE findings of nodular and hard prostates. All the patients responded well to ADT with resolution of the neck masses and reduction in PSA levels. This finding is in keeping with an isolated report of Agbagui *et al.* [10]. The pathogenesis is thought to be lymphatic spread of the prostate malignancy from the hypogastric, obturator, iliac and para-aortic nodes to the supraclavicular lymph nodes. Two patients in addition had lower limb oedema from pelvic lymph node obstruction. Lymphatic involvement is not uncommon in PCa but the absence of LUTS in the presence of supraclavicular swellings makes diagnosis a challenge in these situations. A patient presented with scalp mass which simulated a lipoma. A biopsy of this revealed metastatic adenocarcinoma. There was elevated PSA and abnormal DRE findings. Prostate biopsy confirmed PCa. Isolated cutaneous manifestations of PCa have been reported in literature. Frontal swelling as a manifestation of PCa is however rare in our practice and poses a diagnostic dilemma. This unusual swelling responded well to BTO after PCa diagnosis [11].

## Conclusion

PCa masquerades in many ways and unusual manifestations of PCa should be considered in clinical situations where conventional treatment does not lead to the expected outcome. DRE and PSA should be done in such situations to exclude PCa.

### What is known about this topic

- Prostate cancer has a high disease burden especially in black men and majority of the patients present in advanced disease stage;
- Locally advanced and metastatic PCa can manifest in many bizzare ways, hence a high index of suspicion is required to diagnose this conditions;
- Patients who present with features of advanced disease are only amenable to palliative care by Androgen ablation therapy.

### What this study adds

- This study showcases the various unusual presentation of PCa in our facility;
- It is aimed to educate colleagues these bizzare forms of presentation in order to identify the m early and commence treatment.

## Competing interests

The authors declare no competing interests.

## References

[1] Osegbe DN (1997). Prostate cancer in Nigerians: facts and nonfacts. J Urol.

[2] Salako AA, Arowolo OA, Omonisi EA (2009). Incidental carcinoma of the prostate gland presenting with initial manifestation of disseminated intravascular coagulopathy in a middle-aged man; a case report. Cases Journal.

[3] Eke N, Sapira MK (2002). Prostate cancer in Port Harcourt, Nigeria: features and outcomes. Nig J of Surgical Research.

[4] Bornig CC, Squires TS, Health CWJ (1992). Cancer statistics for African Americans. CA Cancer J Clin.

[5] Shittu OB, Ogunbiyi JO (2003). Orbital metastatsis of prostatic carcinoma in a tropical African Population. West Afr J Med.

[6] Ogunmola AO, Shittu OB, Olapade-Olaopa EO (2013). Cutaneous metastasis from prostate cancer in Nigeria: a case report and literature review. Afr J Med Sci.

[7] Chang DM, Davidson AJ, Sutherland T, De Fontgalland, Johnson D, Wong LM (2017). Unusual presentation of advanced prostate cancer masqyuerading as metastatic and obstructing recto-sigmoid cancer. ANZ J Surg.

[8] Badmus TA, Adesunkanmi ARK, Yusuf BM, Oseni GO, Eziyi KA, Bakare TIB (2010). Burden of prostate cancer in South- Western Nigeria. Urology.

[9] Kabeer Mohammed A, Lloyd-Davies Edward, Maskell Giles (2007). Metastatic prostate cancer masquerading clinically and radiologically as a primary caecal carcinoma. World J Surg Oncol.

[10] Agbagui JO, Obarisiagbon EO, Ugiagbe EE (2014). Advanced carcinoma of the prostate presenting as supraclavicular mass: case report. Niger Postgrad Med J.

[11] Guzman Martinez-Valls PL, Rodriguez TA, Honorubia VB (2009). Frontal mass simulating lipoma as a clinical presentation of prostatic adenocarcinoma. Arch Esp Urol.

